# Synapse Associated Protein 102 (SAP102) Binds the C-Terminal Part of the Scaffolding Protein Neurobeachin

**DOI:** 10.1371/journal.pone.0039420

**Published:** 2012-06-20

**Authors:** Juliane Lauks, Patricia Klemmer, Fatima Farzana, Ramesh Karupothula, Robbert Zalm, Nancy E. Cooke, Ka Wan Li, August B. Smit, Ruud Toonen, Matthijs Verhage

**Affiliations:** 1 Department of Functional Genomics, Center for Neurogenomics and Cognitive Research, Neuroscience Campus Amsterdam, VU University Amsterdam, Amsterdam, The Netherlands; 2 Department of Molecular and Cellular Neurobiology, Center for Neurogenomics and Cognitive Research, Neuroscience Campus Amsterdam, VU University Amsterdam, Amsterdam, The Netherlands; 3 Department of Clinical Genetics, Center for Neurogenomics and Cognitive Research, Neuroscience Campus Amsterdam, VU University Amsterdam and VU Medical Center, Amsterdam, The Netherlands; 4 Department of Genetics and Medicine, Perelman School of Medicine, University of Pennsylvania, Philadelphia, Pennsylvania, United States of America; Virginia Commonwealth University Medical Center, United States of America

## Abstract

Neurobeachin (Nbea) is a multidomain scaffold protein abundant in the brain, where it is highly expressed during development. Nbea-null mice have severe defects in neuromuscular synaptic transmission resulting in lethal paralysis of the newborns. Recently, it became clear that Nbea is important also for the functioning of central synapses, where it is suggested to play a role in trafficking membrane proteins to both, the pre- and post-synaptic sites. So far, only few binding partners of Nbea have been found and the precise mechanism of their trafficking remains unclear. Here, we used mass spectrometry to identify SAP102, a MAGUK protein implicated in trafficking of the ionotropic glutamate AMPA- and NMDA-type receptors during synaptogenesis, as a novel Nbea interacting protein in mouse brain. Experiments in heterologous cells confirmed this interaction and revealed that SAP102 binds to the C-terminal part of Nbea that contains the DUF, PH, BEACH and WD40 domains. Furthermore, we discovered that introducing a mutation in Nbea’s PH domain, which disrupts its interaction with the BEACH domain, abolishes this binding, thereby creating an excellent starting point to further investigate Nbea-SAP102 function in the central nervous system.

## Introduction

Neurobeachin (Nbea), a large (327 kDa), brain-enriched, multi-domain protein is essential for synaptic transmission [1], [2], [3]. Nbea was initially discovered in an attempt to identify novel synaptic proteins, but was subsequently found to associate with tubulovesicular endomembranes near the trans-Golgi network and throughout the neuronal cell body and dendrites [3], [4]. Its membrane association is stimulated by GTP and antagonized by brefeldin A [4]. Hence, Nbea may play a role in post-Golgi sorting or targeting of neuronal membrane proteins and vesicle trafficking [4].

Nbea knock-out (KO) mice lack spontaneous and reflexive movement (i.e. movement elicited by tail pinch) and die immediately after birth due to their inability to breathe [1], [2]. This primary asphyxia is probably the result of the absence of evoked neurotransmitter release at neuromuscular junctions [1]. Also in the central nervous system (CNS) abnormalities in the formation and function of synapses have been described. In fetal Nbea KO brainstem slices, spontaneous and miniature excitatory postsynaptic currents (mini EPSCs) show a reduction in frequency, whereas spontaneous and miniature inhibitory postsynaptic currents (mini IPSCs) are both reduced in frequency and amplitude [2]. Along with a reduced number of asymmetric contacts in the fetal brainstem, reduced levels of several presynaptic proteins were observed [2]. Also, altered miniature excitatory and inhibitory postsynaptic currents were reported in cultured hippocampal neurons from KO mice and cortical slices from heterozygous mice, accompanied by reduced numbers of spine-localized synapses [3]. In addition, in KO neurons excitatory presynaptic terminals are mostly on dendritic shafts instead of on spine heads and actin in these synapses is less enriched [3].

Nbea belongs to a family of BEACH (Beige and Chediak-Higashi) proteins, which share three carboxyl-terminal (C-terminal) domains: a Pleckstrin-Homology like domain (PH) [5], a highly conserved BEACH domain [6] and tryptophan-aspartic acid (WD40) repeats. In addition, some of the BEACH proteins share a domain of unknown function 1088 (DUF 1088).

Pleckstrin-homology domains, first identified as an internal repeat in the phosphoprotein pleckstrin (a substrate of protein kinase C in platelets [7], [8]), comprise a well-defined class of phospholipid-binding protein domains. More than 500 different PH domain-containing proteins have been found [9], many of which are involved in signal transduction and cytoskeleton organization [10]. While generally binding to inositol lipids, a subset of PH domains respond to up-stream signals by targeting the host protein to the correct cellular site [11]. In addition, they can also function in phosphotyrosine binding and mediating protein-protein interaction [5].

The BEACH domain that is adjacent to the PH-like domain is highly conserved among the BEACH proteins (50–60% sequence identity) [12]. With approximately 280 amino acids, it is much larger than a simple protein-protein interaction domain and it has been hypothesized to contain enzymatic activity [4]. However, no catalytic sites have been found [5]. Structural analysis revealed that the BEACH domain is in extensive association with the PH domain [5]. Protein binding assays, using purified PH domain fused with glutathione *S*-transferase and the purified His-tagged BEACH domain of the protein FAN, clearly demonstrated strong interactions between the PH and BEACH domains of FAN [5]. Moreover, specific single-site mutations in FAN’s PH-BEACH interface not only disrupted the interactions between these two domains, but also reduced FAN’s signaling, showing that the two domains may function as a single unit [5].

The WD40 repeat domain (also called the beta-transducin repeat [13] or the GH-WD repeat domain [14]) was first identified in the β-subunit of trimeric G-proteins [15]. A common feature of WD40 repeat domains is that their propeller structures create a stable platform for reversible interactions with multiple other proteins to form complexes [16].

**Figure 1:**
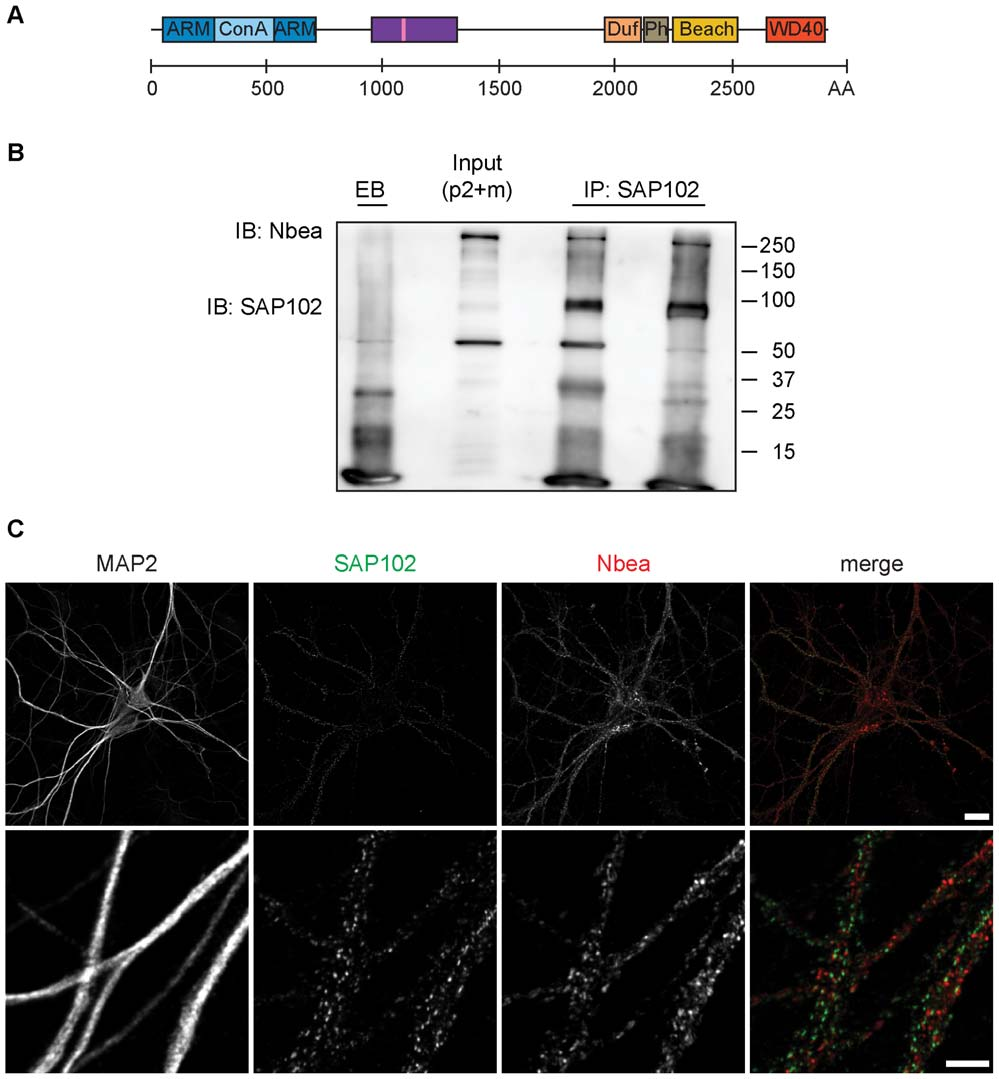
Nbea interacts with a fraction of SAP102 in vivo. (A) Schematic representation of mouse Nbea (NCBI Reference Sequence: NP_085098.1). The predicted armadillo (ARM) repeat-flanked Concavanalin A (Con A)-like lectin domain is localized at the N-terminus of the protein (blue). The region the Nbea antibody was raised against is depicted by the purple rectangle, encompassing also the PKA binding site (pink stripe). At the C-terminus the domain of unknown function 1088 (DUF; in orange), the Pleckstrin-Homology like domain (PH; in gray), the BEACH domain (yellow) and the WD40 repeats (red) are depicted. (B) Co-immunoprecipitation of SAP102 and Nbea from crude membrane with microsomes fraction (P2+M) of P84 WT mice. Proteins were immunoprecipitated (IP) with two different α-SAP102 antibodies, i.e. a mouse monoclonal (NeuroMab clone N19/2; left lane) and a rabbit polyclonal one (GenScript; right lane), respectively. In the control condition non-coated, empty beads (EB) were used for the IP. The *Input* lane represents the crude membrane with microsomes fraction that was used for immunoprecipitation. Immuno-blotting (IB) was performed with α-Nbea and α-SAP102 antibody (NeuroMab clone N19/2). (C) Dendritic Nbea immunoreactivity shows limited overlap with SAP102. DIV14 WT hippocampal neurons (E18) fixed in methanol and stained for endogenous SAP102 (in green), endogenous Nbea (in red) and MAP2 (not shown in the merge). Top scale bar  = 20 µm, lower scale bar  = 5 µm.

The amino-terminal (N-terminal) region of Nbea contains an armadillo repeat-flanked Concavanalin A (ConA)-like lectin domain (Figure 1A) that is shared by other mammalian BEACH proteins, e.g. CHS (Chediak-Higashi syndrome), LRBA and ALFY [17]. Unfortunately, also the function of this domain is still unidentified. Furthermore, apart from the *Drosophila* AKAP550, Nbea is the only BEACH protein with a binding site for the RII regulatory subunit of the 3′5′-cyclic-adenosine-monophosphate (cAMP)-dependent protein kinase (also called Protein kinase A; PKA), classifying it as an A-kinase anchoring protein (AKAP; for graphical representation see Figure 1A) [4], [18]. AKAPs cluster cAMP signaling enzymes in discrete units, creating cAMP microdomains that underlie the spatial and temporal resolution of cAMP signaling [19].

Taken together, Nbea is a complexly organized multidomain protein, and therefore, likely acts as a scaffold for binding of many proteins. So far, only a few binding partners of Nbea have been reported (Table 1) and it has not yet been possible to deduce a role of Nbea in cellular function. In this study we used protein interaction proteomics technology to identify novel Nbea interactors. Here we show that SAP102, a scaffolding protein that has been implicated in trafficking of AMPA and NMDA receptors during synaptogenesis [20], binds to Nbea’s C-terminal part, and that this encompasses the DUF1088, PH, BEACH and WD40 domains. In addition, we describe a mutation in the PH domain that abolishes this binding, creating a solid base to further dissect Nbea-SAP102 function in the CNS.

**Table 1:** Previously identified binding partners of Nbea.

Interactor	Alternative names	Detection method	Reference
PRKAR2A	Protein kinase, cAMP dependent, regulatory type II, alpha	Surface plasmon resonance, pull down	[4]
PRKAR2B	Protein kinase, cAMP dependent, regulatory type II, beta	Surface plasmon resonance, pull down	[4]
BMPR2	Bone morphogenetic protein receptor type II	Pull down	[47]
STRN4	Striatin calmodulin binding protein 4; Zinedin	Two hybrid pooling approach	[48]
Ywhab	Protein kinase C inhibitor protein 1; 14-3-3 protein beta	Co-sedimentation through density gradient	[49]
FYN	Proto-oncogene Syn; Src-like kinase; Proto-oncogene-cFyn; p59-Fyn	Peptide array	[50]
ABL1	Abelson murine leukemia viral oncogene homolog 1; Abelsontyrosine-protein kinase 1; Proto-oncogene c-Abl; p150	Peptide array	[50]
DTNBP1	Dysbindin-1; Hermansky-Pudlak syndrome 7 protein; Dystobrevin-biding protein 1	Two hybrid	[51]
glpD	Y3891; YP_3299; q8cwg4_yerpe	Two hybrid pooling approach	[52]
BA_0681	BAS0647; GBAA_0681; q81v23_bacan	Two hybrid pooling approach	[52]
GlyRB	Glycine receptor beta subunit	Pull down	[21]

## Results

### SAP102 is a Potential Interactor of Nbea

To identify novel interaction partners of Nbea, we performed a proteomics interaction analysis in fetal (embryonic day 18; E18) and adult mice (postnatal day 84; P84) using a α-Nbea antibody, which we generated to immunoprecipitate (IP) potential binding proteins. Characterization of this antibody is shown in Figure S1. Since the Nbea KO mice present a lethal phenotype, we first carried out 3 IPs on E18 brain homogenates. IPs were performed on WT and KO brain homogenates, using α-Nbea antibody (n = 3), and on WT brain homogenates using either beads coated with pre-immune serum (n = 1) or non-coated, empty-beads (EB; n = 1) served as controls for non-specific binding. Immunoprecipitated proteins were separated by SDS-PAGE and visualized by coomassie. Proteins separated on gel, were trypsin digested, and identified by LTQ-Orbitrap mass spectrometry. Table 2 shows the list of proteins that were present in at least 2 of the 3 IP experiments, and were enriched at least 20-fold in the IP samples compared to the controls. In total, 8 proteins were identified, among which the Discs large homolog 3 (Dlg3), also called Synapse Associated Protein 102 (SAP102) was repeatedly identified.

**Table 2:** List of proteins identified from IPs on brain homogenates of E18 mice.

		Unused values		
Protein name	Gene name	Exp 1	Exp 2	Exp3
Neurobeachin	Nbea	204.5	209.2	353.8
Dipeptidyl aminopeptidase-like protein 6	Dpp6	3.7	10	6.1
Discs large homolog 3, Synapse-associated protein 102	Dlg3	6.0	4.2	9.2
Echinoderm microtubule-associated protein like 1	Eml1	2.2	2.0	2.1
Echinoderm microtubule-associated protein like 2	Eml2	5.0	4.6	11.2
Echinoderm microtubule-associated protein like 4	Eml4	2.0	12.2	4.0
Lipopolysaccharide-responsive and beige-like anchor protein	Lrba	/	2.4	2.2
Serine/threonine-protein kinase Nek 9	Nek9	1.7	6.0	2.2

The experiments were performed on brain homogenates of Nbea WT and KO E18 mice using Triton X-100 as detergent for protein extraction. Proteins listed in this table were present in at least 2 of the 3 IP experiments, and were enriched at least 20-fold in the IP samples compared to the controls. The “unused” value is a summation of protein scores from all non-redundant peptides matched to a single protein. Proteins with “unused” value <1.3 have low confidence and were excluded from the analysis.

To examine the Nbea protein interactome in adult mice, we performed IPs on a crude membrane with microsomes fraction (P2+M) from 12 week old mice (n = 3). In addition to the non-coated, empty beads (EB; n = 2), we also used the IP from E18 KO mice as controls. Using the same analysis as for the E18 condition (for a detailed description see Text S1), 29 proteins were identified. Most of the proteins identified from the E18 IP samples were also present in the adult IP samples. Importantly, Dlg3/SAP102 was repeatedly identified (Table 3). In line with this, reverse IP using two different α-SAP102 antibodies on P2+M fractions from adult WT mice, identified Nbea (Figure 1B). These data show that SAP102 and Nbea are part of the same complex in vivo in young (E18), as well as in adult (P84) mice.

**Table 3:** List of proteins identified from IPs on P2+ microsomes fraction from P84 mice.

		Unused values		
Protein name	Gene name	Exp 1	Exp 2	Exp3
**Neurobeachin**	Nbea	108.7	126.1	150.4
**Discs large homolog 3; Synapse-associated protein 102**	Dlg3	2.0	12.1	13.6
Complement component 1Q subcomponent-binding protein, mitochondrial	C1qbp	8.0	6.0	4.0
CAP-Gly domain-containing linker protein 2	Clip2	12.3	4.0	6.0
Dihydrolipoyl dehydrogenase, mitochondrial	Dld	6.0	6.5	6.1
**Dipeptidyl aminopeptidase-like protein 6**	Dpp6	6.0	6.2	9.2
Cytoplasmic dynein 1 intermediate chain 2	Dync1i2	2.0	/	3.7
Elongation factor 1-alpha 1	Eef1a1	4.0	/	6.1
**Echinoderm microtubule-associated protein like 2**	Eml2	/	6.6	6.1
ERC protein 2	Erc2	/	3.6	2.1
Similar to Heat shock protein 1	Gm12141	/	6.0	5.4
Heat shock 70 kDa protein 12A	Hspa12a	16.6	2.0	6.5
Potassium voltage-gated channel subfamily D member 2	Kcnd2	2.4	/	2.0
Similar to hCG45299	LOC100045958	5.7	2.0	5.7
**Serine/threonine-protein kinase Nek 9**	Nek9	/	2.0	2.0
6-Phosphofructokinase type C	Pfkp	3.4	/	2.2
cAMP-dependent protein kinase type II-beta regulatory subunit	Prkar2b	14.4	1.4	/
Protein quaking	Qk	/	4.3	2.3
Mitochondrial glutamate carrier 1	Slc25a22	2.1	2.1	/
Synaptosomal-associated protein 25	Snap25	2.0	2.8	2.0
Sjogren syndrome/scleroderma autoantigen 1 homolog	Sssca1	2.0	1.6	1.4
Striatin	Strn	6.0	2.0	/
Synapsin-2; Synapsin II	Syn2	2.0	2.0	/
Synaptogyrin-1	Syngr1	/	2.0	2.0
TBC1 domain family member 5	Tbc1d5	16.8	/	4.8
Tubulin beta-4A chain	Tubb4a	1.3	3.3	1.3
Cytoplasmic dynein 1 light intermediate chain 2	Dync1li2	/	2.0	4.0
Excitatory amino acid transporter 2	Slc1a2	2.0	2.0	/
Tubulin, alpha-4A chain	Tuba4a, Tuba4	3.3	3.0	4.1

The experiments were performed on P2+ microsomes fraction from P84 WT mice using n-Dodecyl β-D-maltoside (DDM) as detergent for protein extraction. Proteins listed in this table were present in at least 2 of the 3 IP experiments, and were enriched at least 20-fold in the IP samples compared to the controls. The proteins in bold were also identified in IPs performed on brain lysates of E18 Nbea mice. The “unused” value is a summation of protein scores from all non-redundant peptides matched to a single protein. Proteins with “unused” value <1.3 have low confidence and were excluded from the analysis.

### Limited Co-precipitation and Co-localization of SAP102 and Nbea

In comparison to the abundance of identified Nbea peptides (highest unused value 353.8), only a relatively low number of SAP102 peptides (highest unused value 13.6) were identified by mass spectrometry (Table 2 & Table 3). This indicates that only a fraction of SAP102 interacts with Nbea. Vice versa, as shown by the reverse IP (Figure 1B), only a subset of Nbea was immunoprecipitated by SAP102. This notion was further supported by subcellular fractionation (Figure S2A), which revealed that only small amount of both proteins is present in the same fractions, and that the two proteins in general localize to different compartments within a cell. Whereas the largest proportion of SAP102 was found in the postsynaptic density (PSD) fraction, Nbea was mostly enriched in P2, and P2+M fractions, but was also found in synaptosomes. Only a small proportion of Nbea was present in the PSD-enriched fraction.

In line with these findings, Nbea and SAP102 staining patterns show only limited co-localization in DIV14 cultured hippocampal neurons (Figure 1C). Whereas SAP102 was not detectable in the soma, but was only present as puncta in the dendrites, a large fraction of Nbea staining was found in the cell soma. Additionally, we detected a prominent punctate Nbea pattern throughout the dendrites. This is in line with Nbea’s reported localization throughout the cell, especially near the *trans*-Golgi [4], [21].

Nbea did not colocalize with the postsynaptic marker Homer (Figure S2B). This is not surprising, given the fact that Nbea’s immunoreactivity was earlier observed near postsynaptic plasma membranes only in a subset of synapses [4]. Furthermore, some of these were identified as GABAergic synapses between Golgi and granule cells in the cerebellum, and inhibitory synapses of spinal cord neurons [4], [21]. In contrast, we used hippocampal cultures that contain mostly excitatory synapses. An alternative scenario that cannot be ruled out is that Nbea is still present at these postsynaptic sites, but that the epitope is not accessible to the antibody, which would result in lack of immunoreactivity. Similar to previous findings [4], no apparent colocalization with the presynaptic marker VAMP2 could be observed (Figure S3A).

Because SAP102 has been linked to the transport of glutamate receptors [22], we also co-stained GluA1 subunits of AMPA receptors and Nbea (Figure S3B). The α-GluA1 antibody resulted in a diffuse staining pattern throughout the cell without prominent accumulations at the postsynaptic site and no significant overlap with Nbea could be detected. This corresponds with our mass-spectrometry data, where AMPA receptors were never isolated with the α-Nbea antibody in brains of embryonic and adult mice.

Taken together, these data indicate that only a small proportion of SAP102 and Nbea engage in the same complex.

### Nbea and SAP102 Co-immunoprecipitate from Heterologous Cells

**Figure 2:**
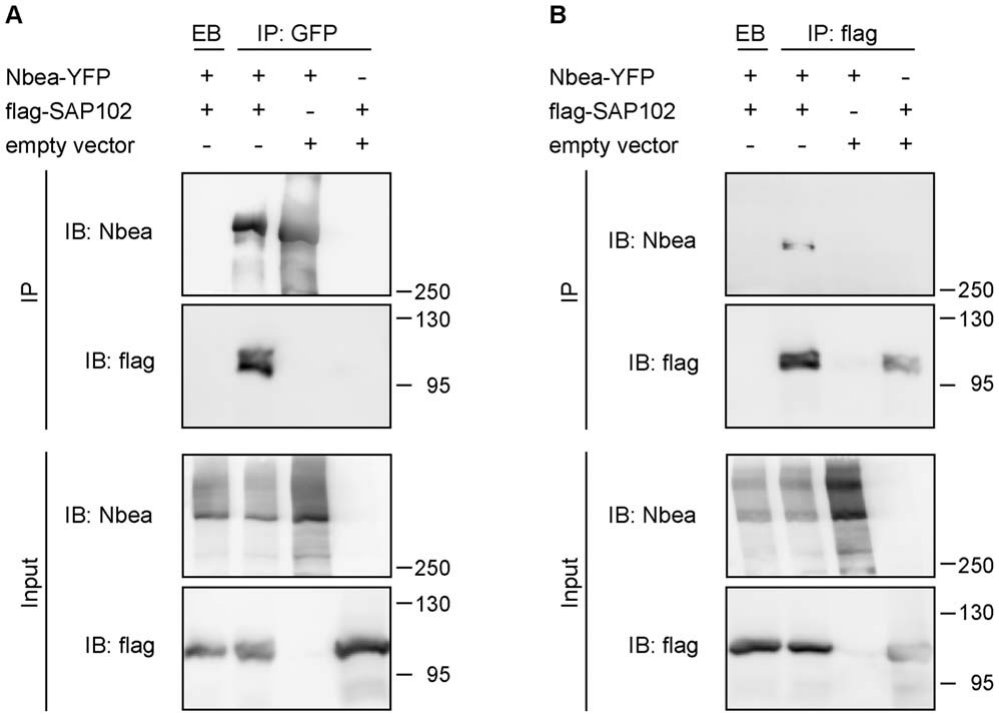
Nbea interacts with SAP102 in HEK293T cells. Co-imunoprecipitation of Nbea and SAP102. (A) HEK 293T cells were co-transfected with full-length Nbea tagged with YFP and flag-tagged SAP102 or an empty vector and were immunoprecipitated (IP) with α-GFP antibody before immuno-blotting (IB) with α-Nbea and α-flag antibody. In the control condition non-coated, empty beads (EB) were used for the IP. (B) Reverse IP to the ones in A. HEK 293T cells were co-transfected with full-length Nbea tagged with YFP and flag-tagged SAP102 or an empty vector, but this time they were immuno-precipitated (IP) with α-flag antibody before immuno-blotting (IB) with α-Nbea and α-flag antibody. In the control condition non-coated, empty beads (EB) were used for the IP.

To further confirm the interaction of SAP102 and Nbea, we performed co-immunoprecipitation (co-IP) assays using heterologous (HEK 293T) cells transfected with DNA constructs expressing full-length Nbea-YFP, FLAG-SAP102 or empty control vector. Αn α-GFP antibody, which also binds YFP, pulled down flag-SAP102 in lysates of cells expressing full-length Nbea, but not in empty vector control (Figure 2A). Although we also used the α-Nbea antibody successfully (see Figure S4C), we decided to use the α-GFP instead. The rationale for this is that the Nbea fragments used in later experiments did not contain the epitope to which the Nbea antibody was raised. Thus, GFP-tagging allowed us to use the same antibody for all co-IPs throughout our study (for additional control experiments see Figure S4). Similarly, in the reverse co-IP assay, α-FLAG pulled down full-length Nbea-YFP in lysates of cells expressing FLAG-SAP102, but not in the empty vector control (Figure 2B). These data suggest that the interaction between Nbea and SAP102 originates either from direct binding or alternatively, that the expressed full-length Nbea-YFP interacts with endogenous HEK cell proteins forming a complex with SAP102 and thereby interacting with SAP102 indirectly.

### SAP102 Binds to the C-terminal Part of Nbea

**Figure 3:**
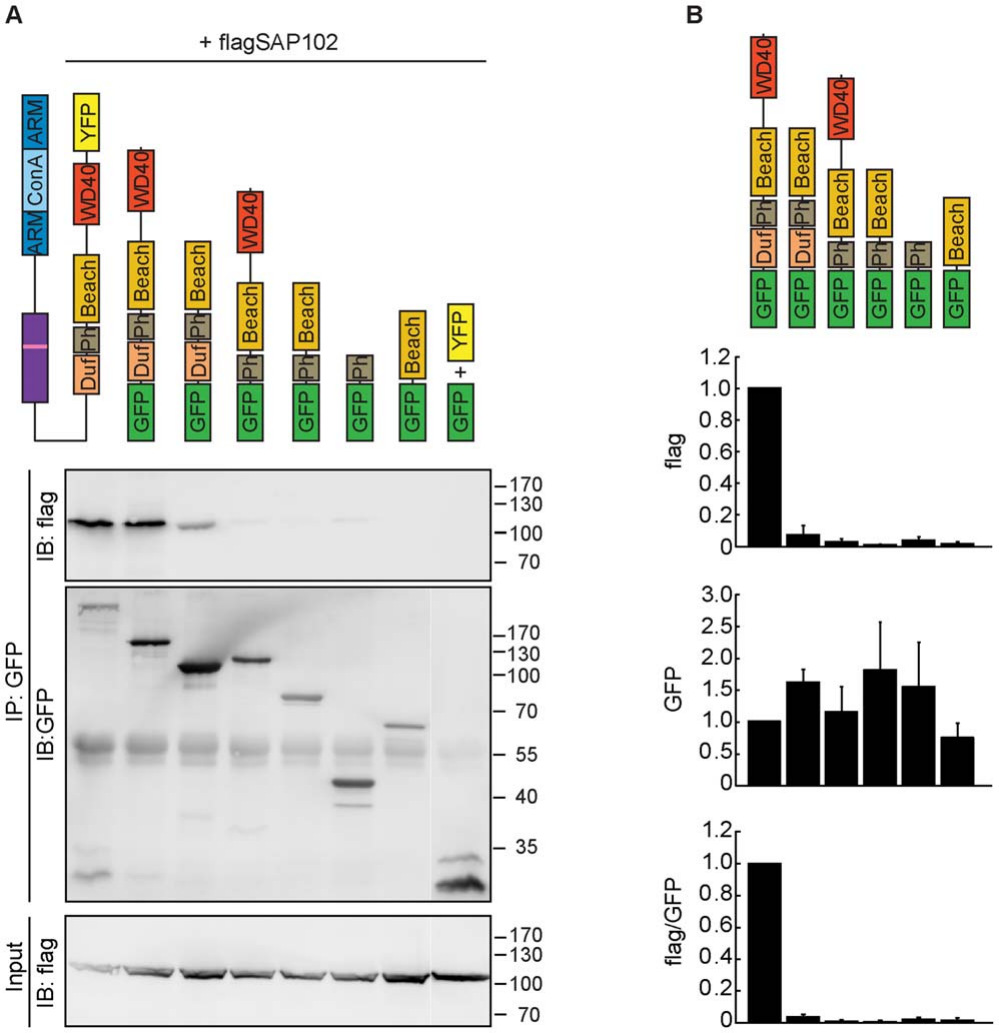
SAP102 binds to the C-terminal part of Nbea. (A) HEK293 cells were co-transfected with flag-tagged SAP102 and either full-length Nbea tagged with YFP or various Nbea deletions encompassing different domains fused to GFP. Following deletions were used: GFP-Duf, PH, BEACH, WD40; GFP-Duf, PH, BEACH; GFP-PH, BEACH, WD40; GFP-PH, BEACH; GFP-PH; GFP-BEACH. In the control condition SAP-102 was co-transfected with YFP and GFP. IPs were performed using the α-GFP antibody, before immune-blotting with α-flag and α-GFP antibodies. (B) Quantification of flag and GFP protein levels using the immuno-labelled bands. Error bars indicate the standard error of the mean (SEM).

To define the domains of Nbea involved in its interaction with SAP102, we transfected HEK293T cells with constructs expressing various deletions of Nbea (Figure 3) along with FLAG-SAP102 and conducted a co-IP assay with α-GFP antibody. In addition to full-length Nbea, only the fragment containing all four domains (DUF, PH, BEACH, WD40) immunoprecipitated SAP102 (Figure 3). As all constructs apart from the full-length lack the N-terminal part of Nbea, the latter does not seem to be a prerequisite for the interaction with SAP102.

Immuno-blot analysis of the input, as well as confocal microscopy (Figure S5) of the transfected HEK cells demonstrated that all constructs were of the expected molecular mass and adequately expressed (Figure 3A, Figure S5). None of the Nbea fragments showed the same subcellular expression pattern as full-length Nbea. Whereas the latter displayed a punctate pattern, the Nbea fragments exhibited an overall diffuse localization, similar to mCherry (Figure S5).

These results imply that SAP102 binds to different domains in Nbea’s C-terminus and that the PH and BEACH domains alone are not sufficient for this interaction. In addition, at least in HEK cells, the N-terminus is necessary for proper localization of full-length Nbea.

### A Mutation within the PH Domain of Nbea Disrupts the Binding to SAP102

To further characterize the Nbea-SAP102 interaction, we engineered mutations in the different domains of Nbea, some of which have previously been analyzed and shown to have functional consequences [5], [23], [24]. The Nbea mutants created included E2090K in the DUF domain (a missense mutation causing a milder form of Chediak-Higashi Syndrome in adults [23]), E2218R in the PH domain and N2302A in the BEACH domain (equivalent mutations in FAN: E256R and N328A, respectively; disrupt the interactions between the PH and BEACH domain [5]), V2346Q, E2447R and P2499S in the BEACH domain and V2773I in the WD40 repeats (corresponds to a missense mutation within the murine LYST gene that causes severe progressive Purkinje cell degeneration [24]; Figure S6).

The sub-cellular expression patterns of the mutated Nbea proteins did not differ from the non-mutated protein, although a fraction of cells also showed a compartmentalized expression of the proteins (Figure S7), which might reflect targeting of these proteins, probably for degradation.

**Figure 4:**
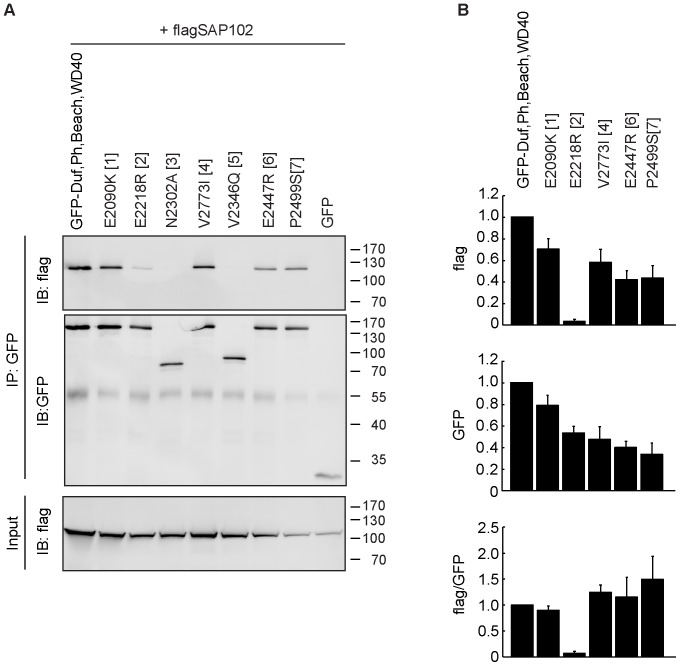
The E2218R mutation within the PH domain abolishes Nbea’s binding to SAP102. (A) HEK293 cells were co-transfected with flag-tagged SAP102 and either the non-mutated C-terminal part of Nbea (encompassing the Duf, PH, BEACH and WD40 domains) fused to GFP or the mutated versions of this protein. The following mutations were used: E2090K in the DUF domain (1), E2218R in the PH domain (2), N2302A in the BEACH domain (3), V2773I in the WD40 domain (4), and an additional three mutations within the BEACH domain, V2346Q (5), E2447R (6) and P2499S (7) (B) Quantification of FLAG-tagged and GFP-tagged protein levels of the immuno-blot. Error bars indicate the standard error of the mean (SEM).

Introducing the E2218R mutation in the PH domain prevented the interaction with SAP102, whereas the other mutations did not show loss of binding (Figure 4B). Thus, either this amino acid is at the interaction interface with SAP102, or more likely, the lost interaction of PH and BEACH domains by this mutation, causes the loss of interaction. This emphasizes the importance of the local orientation of PH and BEACH domains in Nbea with respect to interaction with SAP102.

## Discussion

Nbea was initially identified and characterized as an essential player in synaptic transmission in the peripheral nervous system [1], and further studies confirmed also its vital role in the formation and functioning of central synapses [2], [3]. Considerable evidence has accumulated confirming Nbea’s importance for trafficking cargo to pre-, as well as to post-synaptic compartments [1], [2], [3], [4], [21]. Still, the precise role of Nbea in this has remained unclear. Using an immunoaffinity-based proteomics approach, we identified SAP102 as a novel, Nbea interacting protein in the brains of embryonic and adult mice. Experiments in heterologous cells demonstrated that Nbea bound to SAP102 via the C-terminal part of the protein and that introducing the E2218R mutation in the PH domain disrupted this binding. Because we confirmed this binding by multiple independent approaches, we concluded that Nbea and SAP102 interact.

The Nbea interacting SAP102 is a scaffold protein in excitatory synapses and it belongs to the PSD-95 (post-synaptic density protein of 95 kDa) family of membrane-associated guanylate kinases (MAGUKs). These include in addition to SAP102/Dlg3, also SAP-90 (also known as PSD-95 or Dlg4), PSD93 (also known as Chapsyn-110 or Dlg2) and SAP97 (also known as Dlg1) [25], which all share three PDZ (PSD-95/Discs large/Zona occludens 1) domains, a src-homology 3 (SH3) domain and a C-terminal guanylate kinase (GUK) domain [26]. These large scaffolding proteins are important for clustering and anchoring receptors at the postsynaptic site [25], [27], and through this they can significantly affect synaptic plasticity, i.e. keep or modulate the strength of synaptic transmission between neurons [25].

SAP102 has been shown to associate with NR2A- and NR2B-subunit containing NMDA receptors (NMDARs) in synapses [28], [29], [30], [31], [32]. In addition to its scaffolding function, SAP102 has also been implicated in transport and membrane insertion of NMDA receptors preceding synapse formation [33], [34]. In mice, its expression is highest before P10 and then gradually declines [32]. NR2B receptors exhibit a similar expression pattern [32]. They are trafficked by SAP102 during synaptogenesis [22], before the maturational switch from NR2B- to NR2A-type NMDARs occurs and during synapse maturation when NMDAR trafficking is taken over by PSD95 [22]. Nbea, which also shows a high level of expression during synaptogenesis [4], and SAP102 might be involved in the same pathway of trafficking NMDA receptors.

Only small proportions of the total amount of SAP102 and Nbea engage in the same complex. Since very little Nbea is present in the PSD, where SAP102 is enriched in, it is likely that the interaction of the two proteins takes place predominantly somewhere else in the cell. It has been shown that the MAGUK SAP97 selectively associates with a subset of AMPA receptors early in their biosynthetic pathway [35]. Given Nbea’s localization at the *trans*-Golgi, its enrichment in the P2+M fraction and SAP102’s role in NMDA receptor trafficking, one might speculate that a similar scenario is possible for SAP102 and Nbea, interacting in the early secretory pathway.

Different studies that used expression constructs to dissect the functional domains of BEACH proteins indicate that the various domains can be simultaneously involved in different cellular actions (see [36], [37], [38]), e.g. the PH-BEACH region in Alfy is involved in the direct interaction with the autophagy receptor p62 [39], while the WD40 is essential for its colocalization and interaction with the autophagic marker Atg5 [40].

We found that SAP102 interacts with the C-terminal part of Nbea that contains the DUF, PH, BEACH and WD40 domains. Like the BEACH domain of BGL [41], the BEACH domain of Nbea contains a predicted SH3 binding site. Hence, we expected SAP102 to bind to the BEACH domain and since the PH and BEACH domains might function as a single unit [5], we expected the PH-BEACH fragment to be sufficient for the interaction with SAP102. However, no SAP102 binding was observed when using this construct. This suggests that the DUF and WD40 domains are also necessary for binding or for the correct conformation of the PH-BEACH domains.

We discovered that mutation of E2218R within the PH domain of Nbea compromised SAP102 interaction. This mutation is located within the β6 strand that forms the PH portion of the conserved interface between the PH and BEACH domains together with strands β1, β5 and β7. A similar mutation in FAN disrupts the interaction between these two domains [5], indicating that preservation of this interaction is vital for the association of SAP102.

In addition to SAP102, other interactors of Nbea could also be part of the complex. We identified the RII regulatory subunit of the cAMP-dependent protein kinase (Table 3) in adult mice, confirming earlier findings [4]. Activation of PKA induces synaptic targeting of NMDA receptors [42]. Nbea might also indirectly regulate glutamate receptors via its association with SAP102-NR2B in analogy to the way the AKAP79/150 binds PSD95 and indirectly regulates NMDARs [43].

In our mass spectrometry experiments using young, as well as adult mice, several proteins were identified multiple times, i.e. dipeptidyl aminopeptidase-like protein 6 (DPP6), Echinoderm microtubule-associated protein-like 2 (EML2) and Serine/threonine-protein kinase Nek 9 (Nek9; Table 2 and Table 3). None of these proteins have been linked to Nbea before and might form part of a SAP102/Nbea complex. Further studies will be necessary to reveal their role in this context.

## Materials and Methods

### Ethics Statement

These studies were approved by the institutional ethic committee of the VU University (Protocol FGA 06-11-2). All animals were housed and bred according to the institutional and Dutch governmental guidelines for animal welfare.

Use of human embryonic kidney cells 293T (HEK293T/17; ATCC No: CRL-11268) was approved according to the institutional and Dutch governmental regulations (DGM/RB IG 02-185).

### Laboratory Animals and Cell Lines

Nbea KO mice have been described before [1]. Mouse embryos were obtained at embryonic day 18 by caesarian section of pregnant females from timed mating of Nbea heterozygous animals (C57/Bl6 background). For experiments involving older animals, 12 week old C57/Bl6 mice were used. For mass spec experiments mice were decapitated, the brain was removed and immediately frozen. The tissue was stored at −80°C until further use. For rat neuronal cultures and glia preparations newborn P0–P1 pups from pregnant female Wistar rat (Harlan or Charles River) were used. For immunoprecipitation experiments in heterologous cell lines human embryonic kidney cells 293T (HEK293T) were used.

### Nbea Antibody Production

For the Nbea antibody production a fragment containing the Nbea AA 953-1318 was amplified from the Y2HcDNA library using rz60 5′TGAGGAGTACCAGCGACAAGAGGAG3′ and rz59r 5′CCGAAACATGGTGGTCC3′ and subsequently subcloned into the His-tag vector pQE31 (Qiagen, Hilden, Germany) using SpHI and PstI. In order to create the correct reading frame the construct was digested with SphI, overhangs were modified into blunt ends and selfligated. His-tag fusion protein was expressed in bacteria, purified on nickel agarose, and used for immunization of rabbits. Serum was affinity-purified using the same fusion protein coupled to CNBr-activated Sepharose (GE-Healthcare) according to the manufacturer’s instructions.

### Immunoprecipitation

The fetal brain or P2+microsome fraction from adult mice were solubilized with 1% detergent. For the E18 IPs we employed the commonly used detergent, Triton X-100 for solubilization. In the adult mice brain, especially proteins located in the post-synapse are tightly packed and may be difficult to extract in Triton X-100. We therefore used a stronger detergent, the n-Dodecyl β-D-maltoside (DDM). The extracts were incubated with either 7 µg of the α-Nbea antibody or 10 µg of µ-SAP102 antibody at 4°C on a mechanical rotator. 30 µl slurry of protein A/G PLUS-Agarose beads (Santa Cruz) was washed four times with washing buffer (150 mM NaCl, 25 mM HEPES, 0.1% detergent) before it was added to the samples for 1 h at 4°C.

Afterwards, the beads were washed four times with washing buffer to washout unbound proteins. The buffer was completely removed using an insulin syringe before storing the samples at −20°C until further use.

### Identification of Proteins from IP Samples

The ID PAGE LC-MS/MS approach was used for protein identification as described previously ([44], Text S1). In short, after separation on the SDS PAGE gel proteins were trypsin digested. The resulting peptides were separated on a capillary C18 column using a nano LC-ultra 1D plus HPLC system (Eksigent), and analyzed on-line with an electrospray LTQ-Orbitrap Discovery mass spectrometer (Thermo Fisher Scientific). MS/MS spectra were searched against a mouse database (uniprot_sprot_101020) with the ProteinPilot™ software (version 3.0; AB-Sciex) using the Paragon™ algorithm (version 3.0.0.0; [45]) as the search engine. The search parameters were set to cysteine modification by acrylamide and digestion was done with trypsin. The detected protein threshold (unused protscore (confidence)) in the software was set to 0.10 to achieve 20% confidence, and identified proteins were grouped to minimize redundancy.

Proteins with “unused” value <1.3 have low confidence and were excluded from the analysis. The “unused” value is defined in the handbook of ProteinPilot as a summation of peptide scores from all the non-redundant peptides matched to a protein. Peptides with confidence of ≥99% would have a peptide score of 2; ≥95% a peptide score of 1.3, and ≥66% a peptide score of 0.47, etc. Tryptic peptides shared by multiple proteins will be assigned to the winner protein.

### Plasmids and Generation of Nbea Constructs

The full-length Nbea was generated by using a yeast-two-hybrid cDNA library (Clontech CAT# ML408AH) and a partial image clone (Kazusa mKIAA1544). First, the N-terminal part of Nbea was obtained from the yeast-two-hybrid cDNA library and subcloned in pCR-Script (Stratagene Cat# 211190) using the following primers:

rz62 5′TGCACAGCTCCTCAGCAGCG3′; rz63r 5′GCTGGGTGTTCTGACATTAGAGCC3′ and rz64 5′CAGCTCATATTAAAGGATCGAGG3′; rz65r 5′GGATGAGGGATAGATGGTATGACC3′. The resulting subclones were ligated at PstI and ScaI sites. Then, the C-terminal part from the Kazusa image clone was connected to the N-terminal part using NotI and SpeI resulting in a full-length Nbea in a pCR-Script backbone. A fusion of EYFP and Nbea was made by digesting the Nbea full-length pCR-Script with SalI & KspI and ligating this into the pEYFP-C1, digested with the same enzymes.

For creating the EGFP-Nbea C-terminal fusion construct (containing the DUF1088, PH, BEACH and WD40 domains) the Kazusa image clone was used as template and a C-terminal Nbea fragment containing AA 1956 - 2936 was amplified using rz106 5′AAAGAATTCACCATGGCGGAAGGAAGGTTGTTGTGCCATGC3’ (adding an EcoRI site) and rz118r 5′TTTGGATCCCACTTGAATGTGGCTTCTGCTGC3’ (adding a BamHI site), which was subcloned into pCR-Script. EcoRI and BamHI sites were used for cloning into pEGFP-C3 (See Text S1 on how the rest of the truncations were created).

To create point mutations in the C-terminus of Nbea the standard protocol of QuickChange™Site-Directed Mutagenesis Kit was used. The mutated inserts were subsequently cloned in expression vector and fused to GFP. For information on primers used to create the point mutations see Text S1.

The FLAG-tagged SAP102 was obtained from the yeast-two-hybrid cDNA library, by using the following primers to create mouse SAP102 with 5′EcoRV and 3′SalI restriction sites: 5′GATATCATGCACAAGCACCAGCACTGCTGTAAG3’ and 5′GTCGACTCAGAGTTTTTCAGGGGATGGGACCCA3’. It was first cloned into a pCR-blunt vector (Invitrogen), before it was subcloned, using the EcoRV and SalI sites, into a pCMV3TAG1A vector (Invitrogen). pEGFP and pmCherry were both purchased from Clontech (CAT#PT2039-5, CAT#PT3973-5). All created constructs were sequence-verified.

### Construct Expression and Co-imunoprecipitation in HEK Cells

For expression of DNA constructs, HEK293T cells were cultured in DMEM medium (Invitrogen) containing 10% fetal calf serum (FCS), 1% non-essential amino acids (NEAA) and 1% penicillin/streptomycin (all Gibco)**.** They were plated at equal density in 10 cm dishes one day before transfection. Cells were transfected with calcium phosphate transfection (for details see Text S1) at 80% confluence and grown for 41 hrs after transfection before they were lysed in 800 µl of lysis buffer (containing 50 mM Tris pH 7.5, 1% Triton X-100, 1.5 mM MgCl_2_, 5.0 mM EDTA, 100 mM NaCl, protease inhibitor). After centrifugation at 14000 rpm for 10 min at 4°C, the supernatant was used for immunoprecipitation assays. The lysates were precleared of immunoglobulin by incubation for 1 h at 4°C with Protein A Agarose beads (Sigma). The latter were washed three times with lysis buffer before usage and were afterwards removed by centrifugation. The precleared cell lysates were then incubated with Protein A Agarose beads that were preblocked in 1% chicken egg albumin (Sigma) and were removed after incubation with cell lysate by centrifugation. In the different co-imunoprecipitations we either added the α-Nbea antibody (rabbit polyclonal, 0.18 µg), the α-GFP antibody (rabbit polyclonal, Abcam ab290/50, 0.25 µg) or the α-FLAG antibody (mouse monoclonal clone M2, Sigma, 1.25 µg) for 2 h at 4°C. Afterwards the beads were washed five times with lysis buffer and resuspended in SDS-PAGE loading buffer, and samples were subjected to gel electrophoresis and immuno-blotting. In short, samples were loaded onto 5%–10% SDS-PAGE gels and run on 25 mA per gel until satisfactory mass separation. Proteins were then transferred to PVDF membranes (Bio-Rad) at 350 mA for 2 hrs. Blocking with 2% milk (Merck) and 0.5% bovine serum albumin (BSA; Sigma) for 1 h was used to circumvent unspecific binding. Primary antibodies (α-Nbea rabbit polyclonal, SySy, 1∶500; α-GFP rabbit polyclonal, Abcam, 1∶5000 and α-FLAG mouse monoclonal clone, Sigma, 1∶5000) were applied for 2 hrs or overnight at 4°C. After substantial washing, alkaline phosphatase labeled secondary antibodies (goat α-mouse AP 1∶10000 or goat α-rabbit AP 1∶10000; both DAKO) were applied for 1 h at 4°C. Blots were then washed and scanned using ECF substrate for immuno-blot (GE Healthcare) on a Fujifilm FLA-5000 Reader. All solutions for blocking, staining or washing were prepared in PBS (pH 7.4) containing 0.1% Tween-20 (Sigma). Immuno-blots were stripped using Re-blot Plus Strong Antibody Stripping Solution (Millipore).

### Immuno-blot Data Analysis

For quantification of immuno-blot band intensities the GelAnalyzer tool in ImageJ (NIH; Bethesda, MD) was used. The bar graphs in Figure 3B and Figure 4B depict average intensities of at least 3 separate experiments. Since there might be a difference in the affinity of the α-GFP antibody for GFP and YFP, we did not decide to normalize the data to the full-length Nbea fused to YFP. Instead, we normalized it to the intensity of the band obtained with the C-terminal domain fragment (encompassing the Duf, PH, BEACH and WD40 domain) fused to GFP.

### Dissociated Hippocampal Cultures and Immunofluorescence Staining

Hippocampi were dissected from embryonic day 18 (E18) wild type C57/Bl6 mice and collected in ice-cold Hanks Buffered Salt Solution (HBSS; Sigma), buffered with 7 mM HEPES (Invitrogen). They were incubated in Hanks-HEPES with 0.25% trypsin (Invitrogen) for 20 min. at 37°C. After washing, neurons were triturated using a fire-polished Pasteur pipette and counted in a Fuchs-Rosenthal chamber. The cells were plated in pre-warmed Neurobasal medium (Invitrogen) supplemented with 2% B-27 (Invitrogen), 1.8% HEPES, 0.25% glutamax (Invitrogen) and 0.1% Pen/Strep (Invitrogen) at a density of 25 k/well on 18 mm glass coverslips and allowed to grow for 14 days before fixation.

For the characterization of the α-Nbea antibody, neurons were plated on glass coverslips coated with 30 µg/ml poly-L-lysine (Sigma) and 2 µg/ml laminin (Sigma) in Dulbecco’s Phosphate Buffered Saline (D-PBS, Gibco). On DIV14 neurons were fixed in 4% paraformaldehyde (Sigma) in D-PBS for 20 min, before being rinsed with D-PBS. Subsequently, they were permeabilized for 5 min in D-PBS containing 0.5% Triton X-100, followed by a 30 min incubation in D-PBS containing 0.1% Triton X-100 and 2% normal goat serum to block aspecific binding. The same solution was used for diluting antibodies. Cells were incubated for 2 hrs in the primary antibody mixture containing α-Nbea (rabbit polyclonal, SySy, 1∶1000) and α-MAP2 (mouse monoclonal AP20, Chemicon 1∶1000), washed 3 times with D-PBS and incubated in corresponding secondary antibodies for 1 h. The latter consisted of goat α-mouse Alexa Fluor 543 and goat α-rabbit Alexa Fluor 647 (Molecular Probes, 1∶1000).

For colocalization experiments neurons were plated on coverslips, coated with a mixture of 0.1 mg/ml poly-D-lysine (Sigma), 0.2 mg/ml rat tail collagen (BD Biosciences) solution and 10.2 mM acetic acid solution (Sigma), containing a glial feeder layer. At DIV14-15 they were fixed either in 100% methanol (Interchema) for 4 min (for co-staining with α-SAP102) or in 3.7% formaldehyde (Electron Microscopy Sciences) in D-PBS (for co-stainings with Homer1, VAMP2 and GluA1) for 20 min before being rinsed with D-PBS. They were incubated for 2 hrs in the primary antibody mixture containing α-Nbea (rabbit polyclonal, SySy, 1∶1000), α-MAP2 (chicken polyclonal, Abcam ab5392, 1∶10000), combined with either α-SAP102 (mouse monoclonal, NeuroMab clone N19/2, 1∶100) or α-Homer1 (mouse monoclonal SySy clone 2G8, 1∶250) or α-VAMP2 (mouse monoclonal, SySy clone 69.1, 1∶1000) or GluA1 (mouse monoclonal, SySy clone 160E5, 1∶200), diluted in D-PBS, washed 3 times with D-PBS and incubated in corresponding secondary antibodies (all Molecular Probes, 1∶1000) diluted in D-PBS for 1 h. After additional 3 washes they were mounted on microscopic slides with ProLong®Gold (Invitrogen) and imaged with a 63X Plan-Neofluar lens (Numerical aperture 1.4, Carl Zeiss b.v. Weesp) on a Zeiss 510 Meta Confocal microscope (Carl Zeiss).

## Supporting Information

Figure S1 **Nbea antibody specificity.** (A) Immunostaining of endogenous Nbea and MAP2 in DIV14 WT, heterozygous and KO hippocampal neurons plated on poly-D-lysine/laminin (Sigma). Scale bar  = 5 µm. (B) Immunoblots of whole brain homogenates from Nbea heterozygous, WT and KO mice, probed with α-Nbea antibody.
(TIF)

Figure S2 **Subcellular localization of Nbea and SAP102.** (A) Hippocampi from adult WT mice were used to obtain different subcellular fractions (see Text S1), which were analyzed by immuno-blotting for the presence of Nbea (using a rabbit polyclonal antibody; SySy, 1∶1000), SAP102 (using a mouse monoclonal antibody; NeuroMab clone N19/2, 1∶1000) and PSD-95 (using a rabbit polyclonal antibody; Genescript, 1∶1000). (B) DIV14 rat hippocampal neurons transfected via calcium phosphate transfection at DIV10 with EGFP (not shown in the merge). The calcium-phosphate-mediated method was described previously [46]. After fixation cells were co-stained for the postsynaptic marker Homer1 with a mouse monoclonal antibody (in green; SySy clone 2G8, 1∶250) and Nbea with a rabbit polyclonal antibody (in red; SySy, 1∶1000). We decided to use neurons of rat instead of mouse, because the spines are more prominently observed in rat neurons. Top scale bar  = 20 µm, lower scale bar  = 5 µm.
(TIF)

Figure S3 **Nbea does not localize to the pre-synapse and shows only little overlap with GluA1.** (A) DIV15 WT mouse hippocampal neurons (E18) stained for VAMP2 (in green), Nbea (in red) and MAP2 (not shown in the merge). Top scale bar  = 20 µm, lower scale bar  = 5 µm. (B) DIV14 WT mouse hippocampal neurons (E18) stained for GluA1 (in green), Nbea (in red) and MAP2 (not shown in the merge). Top scale bar  = 20 µm, lower scale bar  = 5 µm.
(TIF)

Figure S4 **Control IPs confirming the interaction of Nbea and SAP102 in HEK293T cells.** (A) Co-imunoprecipitation of Nbea and SAP102. HEK 293T cells were co-transfected with full-length Nbea tagged with YFP and flag-tagged SAP102 or an empty vector and were immuno-precipitated (IP) with α-GFP antibody before immuno-blotting (IB) with α-GFP and α-flag antibody. In the control condition non-coated, empty beads (EB) were used for the IP. (B) Reverse IPs of IPs performed in A. This time the α-flag antibody was used for IPs, while the same antibodies were used for immuno-blotting. (C) HEK 293 cells were co-transfected with full-length Nbea tagged with YFP and flag-tagged SAP102 or an empty vector and were immuno-precipitated (IP) with α-Nbea antibody before immuno-blotting (IB) with α-Nbea and α-flag antibody. (D) Reverse IP of IPs performed in C. This time the α-flag antibody was used for IPs, while the same antibodies were used for immuno-blotting.
(TIF)

Figure S5 **Subcellular localization of Nbea deletion constructs in HEK293T cells.** HEK293T cells co-transfected via calcium transfection with either full-length Nbea-YFP or different GFP-fused Nbea deletions and mCherry. Scale bar  = 5 µm.
(TIF)

Figure S6 **The mutations introduced in the** **C-terminal amino acid sequence of Nbea.** The shaded areas represent the amino acid sequence of the domain of unknown function 1088 (DUF; in orange), the Pleckstrin-Homology like domain (PH; in gray), the BEACH domain (yellow) and the WD40 repeats (red). The red squares depict the amino acids that have been mutated in our study. The numbers on top of the squares are used for identification of the mutations (see also Figure 4 and Figure S7).
(TIF)

Figure S7 **Subcellular localization of Nbea mutation constructs in HEK293T cells.** (A) HEK293 cells co-transfected via calcium transfection with either the non-mutated form of the C-terminal part of Nbea (encompassing the Duf, PH, BEACH and WD40 domains) fused to GFP or the mutated versions of this construct and mCherry (not shown). Scale bar  = 5 µm. (B) Quantification of the proportion of cells exhibiting a compartmentalized pattern. Error bars indicate the standard error of the mean (SEM).
(TIF)

Text S1 **Supporting information.**
(DOC)
