# Supplementary figures and images for: Synapse Associated Protein 102 (SAP102) Binds the C-Terminal Part of the Scaffolding Protein Neurobeachin
Source: PLoS One. 2012 Jun 20;7(6):e39420. doi: 10.1371/journal.pone.0039420 (PMC3380004; doi:10.1371/journal.pone.0039420)

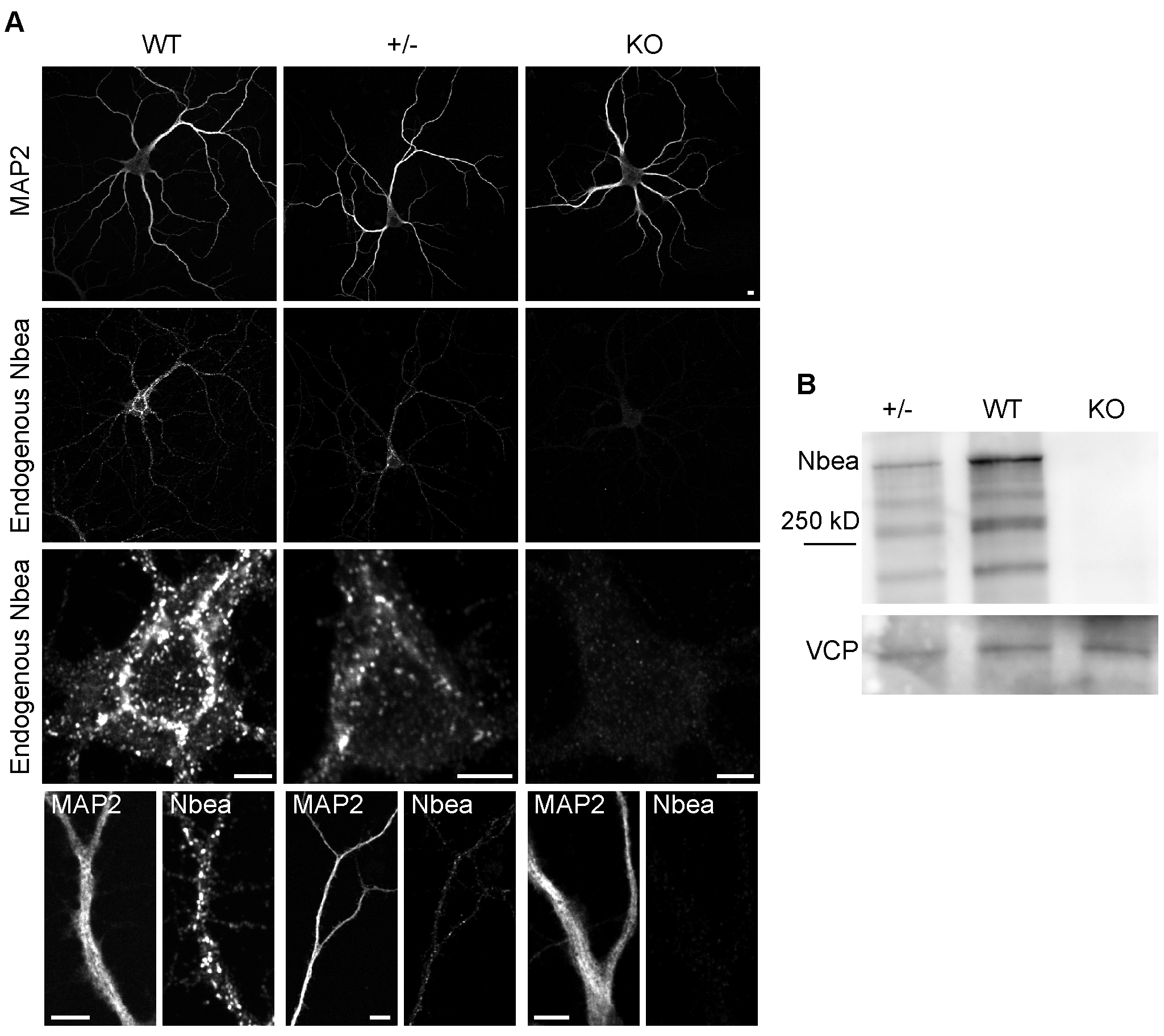

Supplement: Figure S1 — Nbea antibody specificity. (A) Immunostaining of endogenous Nbea and MAP2 in DIV14 WT, heterozygous and KO hippocampal neurons plated on poly-D-lysine/laminin (Sigma). Scale bar = 5 µm. (B) Immunoblots of whole brain homogenates from Nbea heterozygous, WT and KO mice, probed with α-Nbea antibody. (TIF) [file pone.0039420.s001.tif]

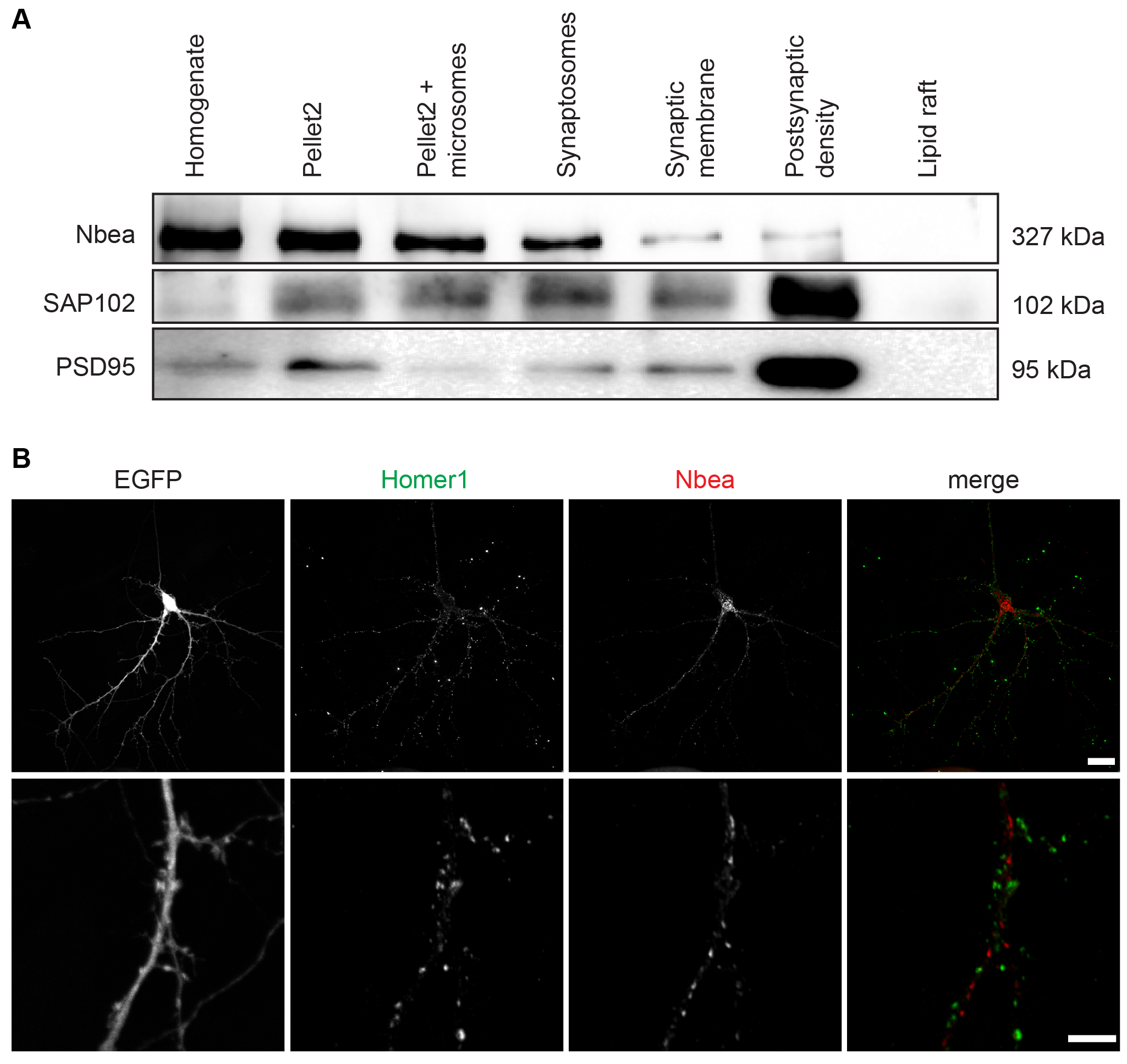

Supplement: Figure S2 — Subcellular localization of Nbea and SAP102. (A) Hippocampi from adult WT mice were used to obtain different subcellular fractions (see Text S1), which were analyzed by immuno-blotting for the presence of Nbea (using a rabbit polyclonal antibody; SySy, 1∶1000), SAP102 (using a mouse monoclonal antibody; NeuroMab clone N19/2, 1∶1000) and PSD-95 (using a rabbit polyclonal antibody; Genescript, 1∶1000). (B) DIV14 rat hippocampal neurons transfected via calcium phosphate transfection at DIV10 with EGFP (not shown in the merge). The calcium-phosphate-mediated method was described previously [46]. After fixation cells were co-stained for the postsynaptic marker Homer1 with a mouse monoclonal antibody (in green; SySy clone 2G8, 1∶250) and Nbea with a rabbit polyclonal antibody (in red; SySy, 1∶1000). We decided to use neurons of rat instead of mouse, because the spines are more prominently observed in rat neurons. Top scale bar = 20 µm, lower scale bar = 5 µm. (TIF) [file pone.0039420.s002.tif]

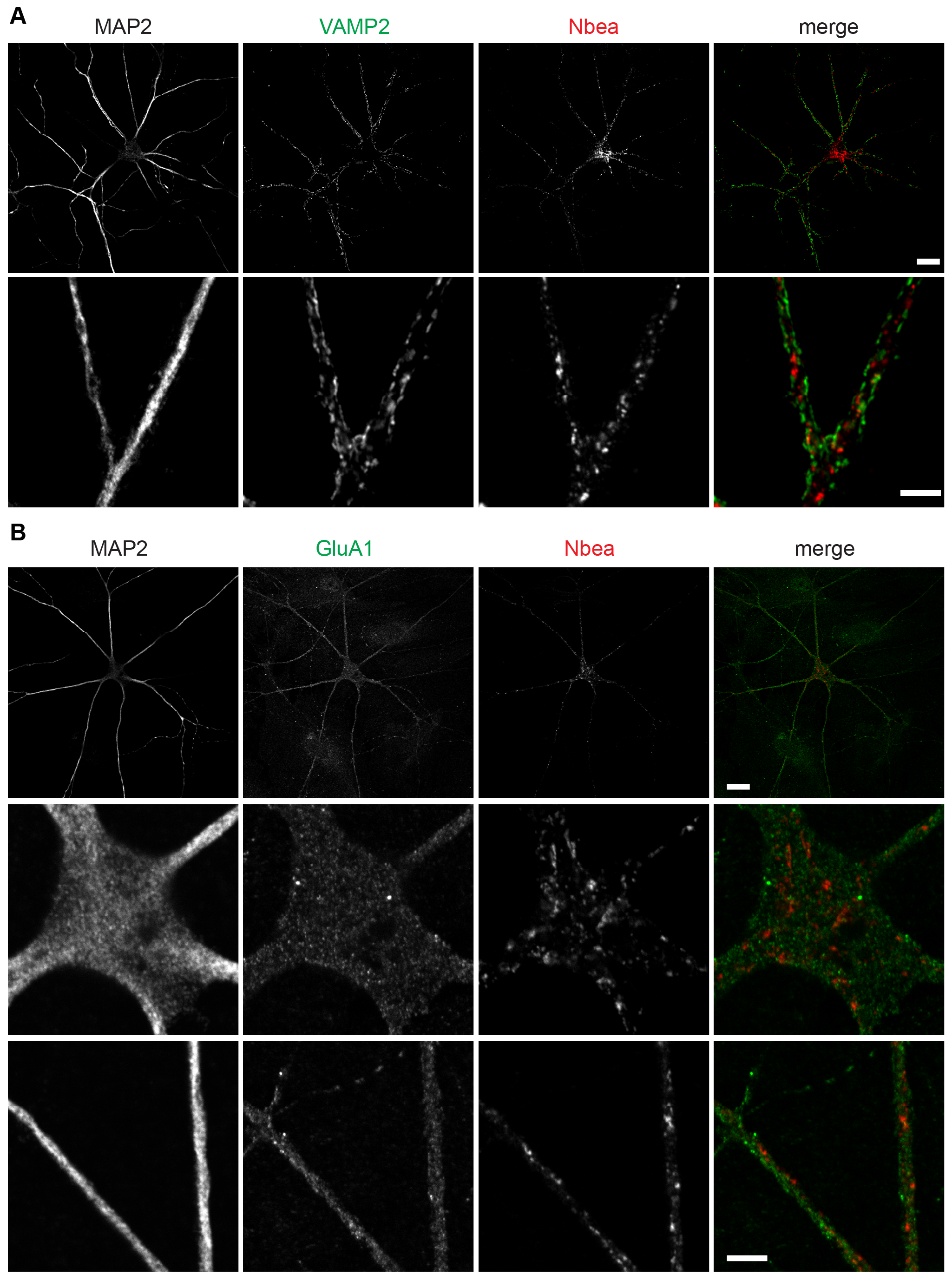

Supplement: Figure S3 — Nbea does not localize to the pre-synapse and shows only little overlap with GluA1. (A) DIV15 WT mouse hippocampal neurons (E18) stained for VAMP2 (in green), Nbea (in red) and MAP2 (not shown in the merge). Top scale bar = 20 µm, lower scale bar = 5 µm. (B) DIV14 WT mouse hippocampal neurons (E18) stained for GluA1 (in green), Nbea (in red) and MAP2 (not shown in the merge). Top scale bar = 20 µm, lower scale bar = 5 µm. (TIF) [file pone.0039420.s003.tif]

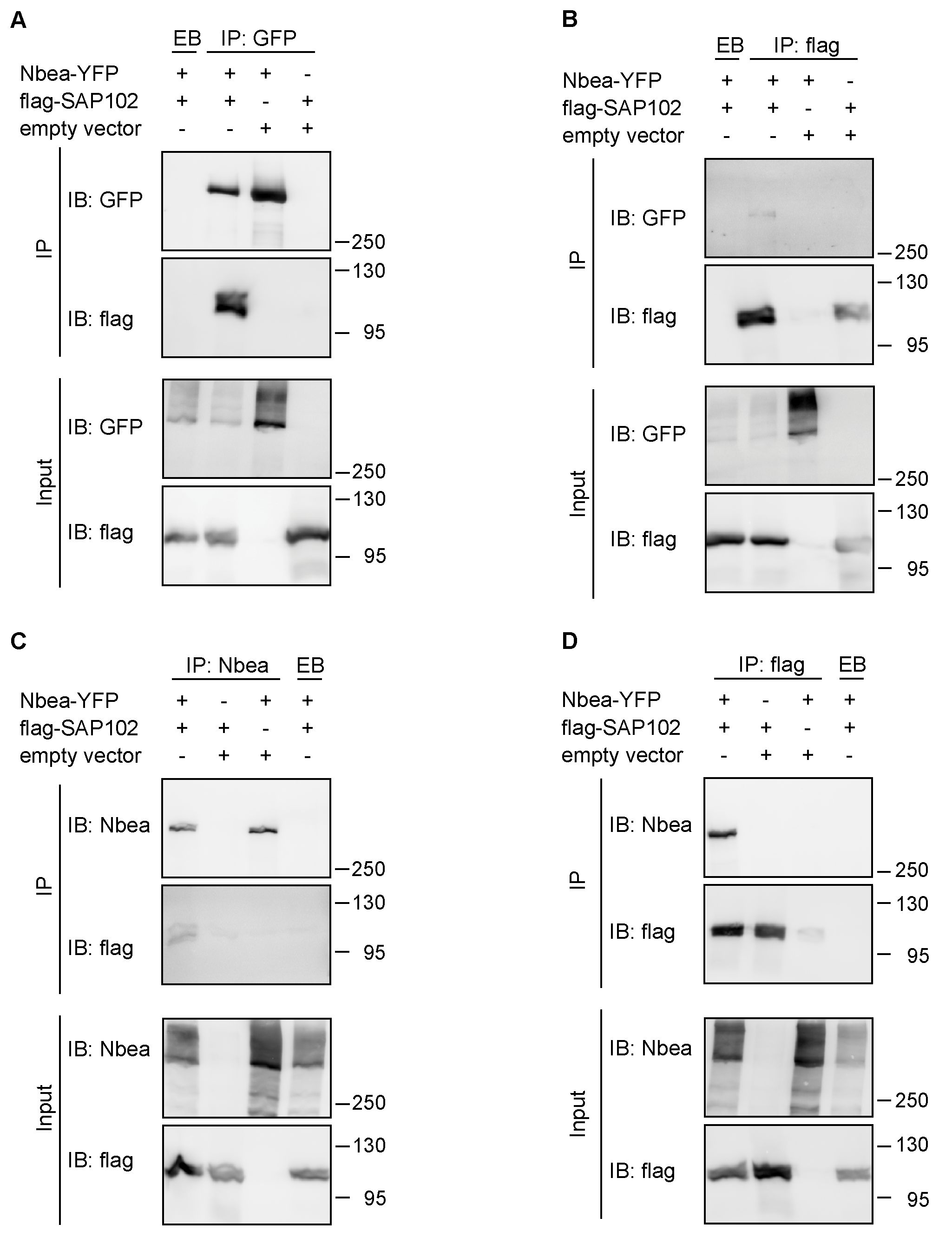

Supplement: Figure S4 — Control IPs confirming the interaction of Nbea and SAP102 in HEK293T cells. (A) Co-imunoprecipitation of Nbea and SAP102. HEK 293T cells were co-transfected with full-length Nbea tagged with YFP and flag-tagged SAP102 or an empty vector and were immuno-precipitated (IP) with α-GFP antibody before immuno-blotting (IB) with α-GFP and α-flag antibody. In the control condition non-coated, empty beads (EB) were used for the IP. (B) Reverse IPs of IPs performed in A. This time the α-flag antibody was used for IPs, while the same antibodies were used for immuno-blotting. (C) HEK 293 cells were co-transfected with full-length Nbea tagged with YFP and flag-tagged SAP102 or an empty vector and were immuno-precipitated (IP) with α-Nbea antibody before immuno-blotting (IB) with α-Nbea and α-flag antibody. (D) Reverse IP of IPs performed in C. This time the α-flag antibody was used for IPs, while the same antibodies were used for immuno-blotting. (TIF) [file pone.0039420.s004.tif]

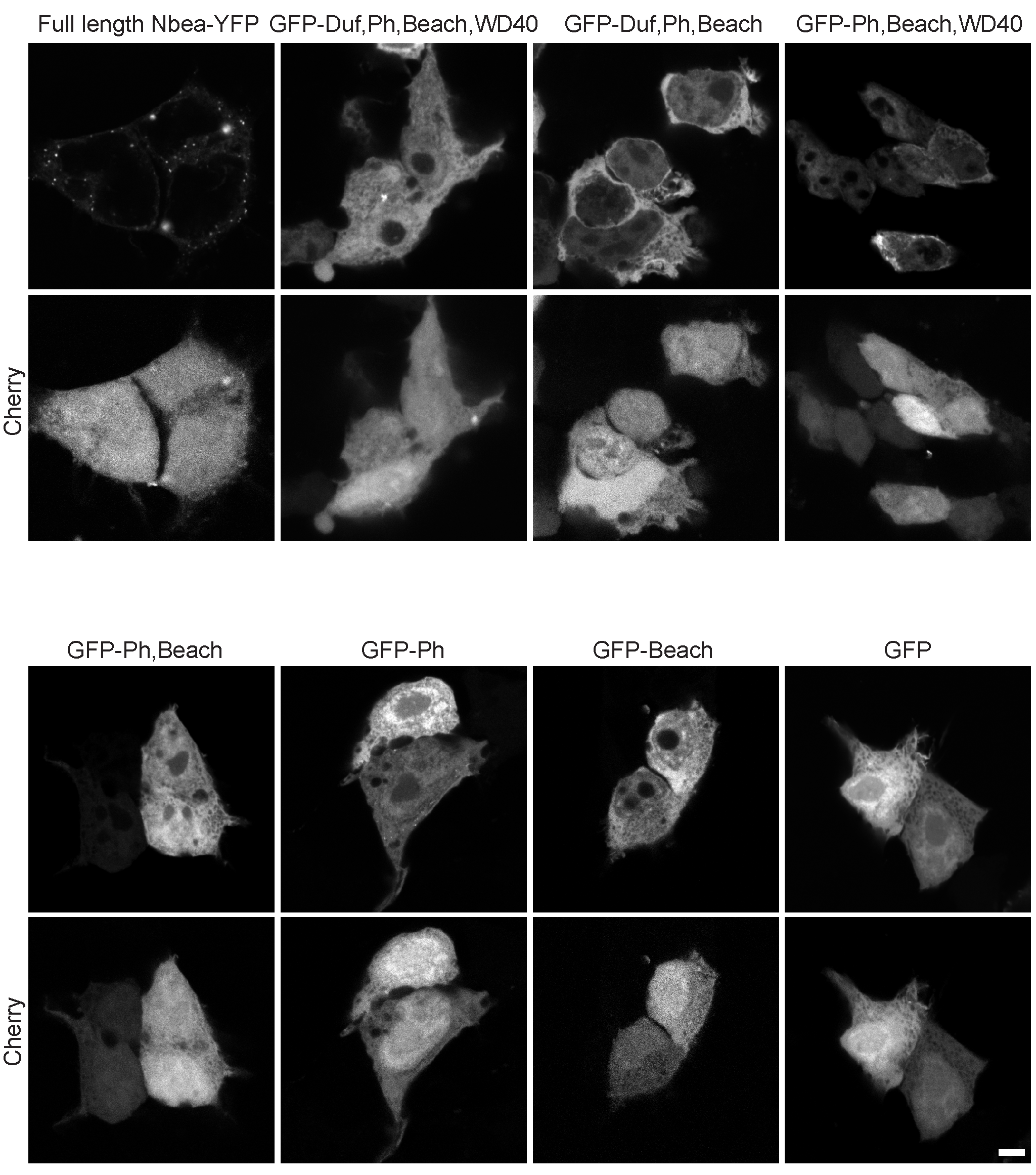

Supplement: Figure S5 — Subcellular localization of Nbea deletion constructs in HEK293T cells. HEK293T cells co-transfected via calcium transfection with either full-length Nbea-YFP or different GFP-fused Nbea deletions and mCherry. Scale bar = 5 µm. (TIF) [file pone.0039420.s005.tif]

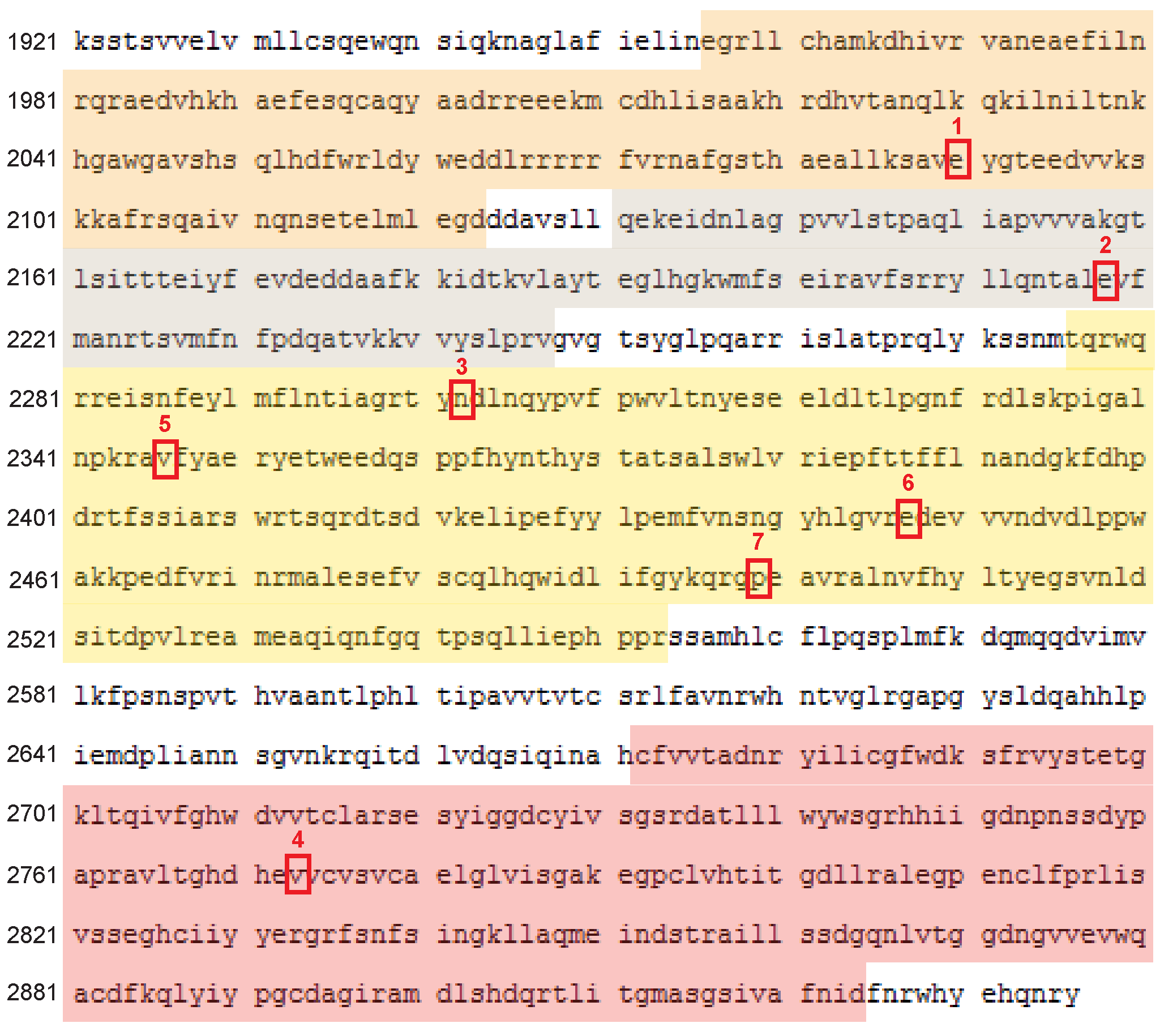

Supplement: Figure S6 — The mutations introduced in the C-terminal amino acid sequence of Nbea. The shaded areas represent the amino acid sequence of the domain of unknown function 1088 (DUF; in orange), the Pleckstrin-Homology like domain (PH; in gray), the BEACH domain (yellow) and the WD40 repeats (red). The red squares depict the amino acids that have been mutated in our study. The numbers on top of the squares are used for identification of the mutations (see also Figure 4 and Figure S7). (TIF) [file pone.0039420.s006.tif]

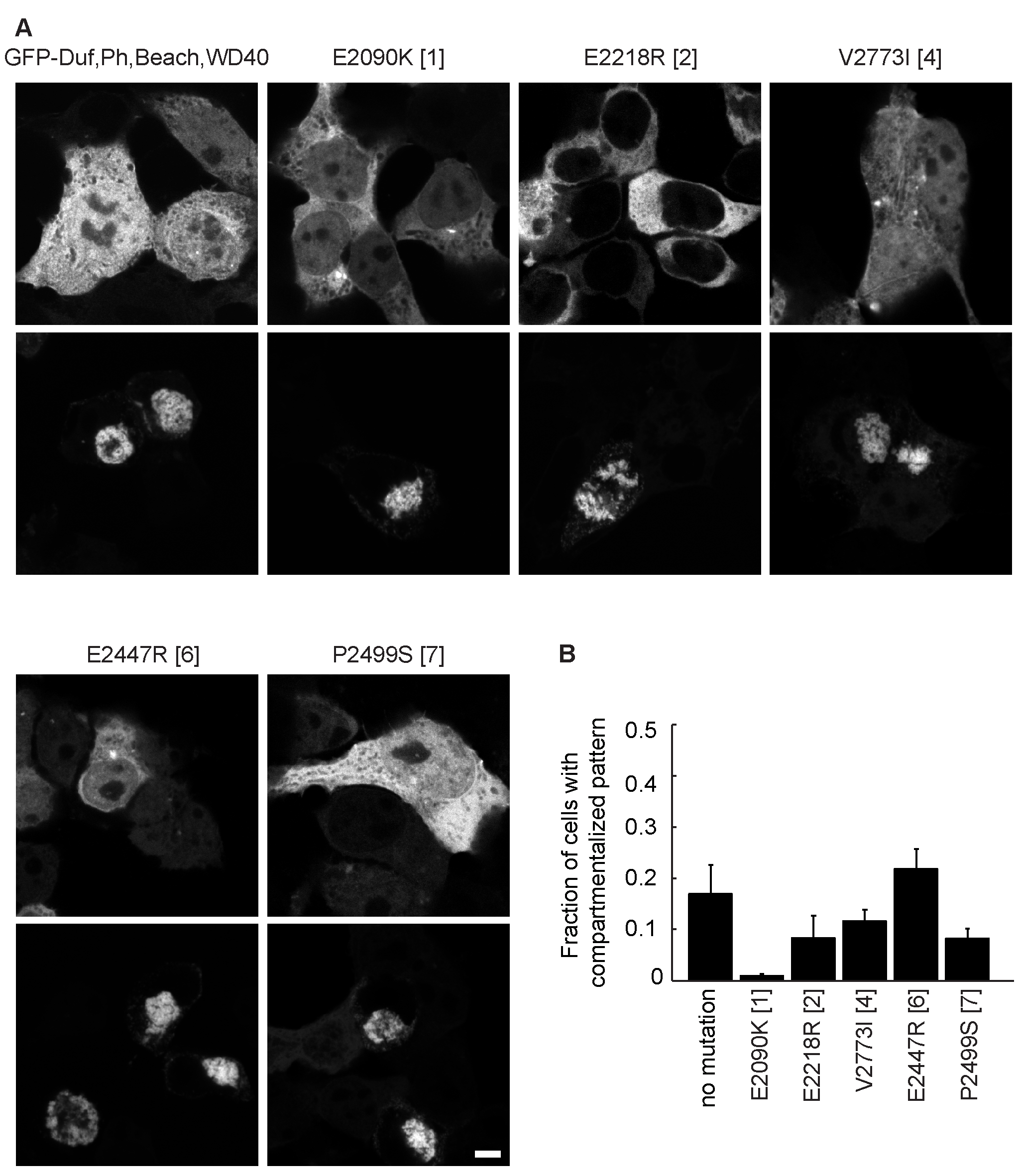

Supplement: Figure S7 — Subcellular localization of Nbea mutation constructs in HEK293T cells. (A) HEK293 cells co-transfected via calcium transfection with either the non-mutated form of the C-terminal part of Nbea (encompassing the Duf, PH, BEACH and WD40 domains) fused to GFP or the mutated versions of this construct and mCherry (not shown). Scale bar = 5 µm. (B) Quantification of the proportion of cells exhibiting a compartmentalized pattern. Error bars indicate the standard error of the mean (SEM). (TIF) [file pone.0039420.s007.tif]
