# Supplementary material for: Synapse Associated Protein 102 (SAP102) Binds the C-Terminal Part of the Scaffolding Protein Neurobeachin
Source: PLoS One. 2012 Jun 20;7(6):e39420. doi: 10.1371/journal.pone.0039420 (PMC3380004; doi:10.1371/journal.pone.0039420)
Supplement: Text S1 — Supporting information. (DOC) [file pone.0039420.s008.doc]

**Supporting** **information**

**Mass spectrometry**

Preparation of brain lysates

For immuno-precipitation Triton X-100 (TX-100) or n-Dodecyl β-D-maltoside (DDM) extracts were prepared by adding extraction buffer (25 mM HEPES pH7.4, 150 mM NaCl, 1% detergent, protease inhibitor (complete Mini, EDTA-free; Roche Applied Science)) to E18 WT or Nbea KO mouse brains followed by homogenization at 900 rpm on ice using a Potter homogenizer (B. Braun Biotech International). We used 2 brains per IP and 1ml of extraction buffer per brain. The homogenate was then left to slowly rotate for 20 min at 4°C, before it was centrifuged at 20000 rcf for 20 min at 4°C. Afterwards, the supernatant was transferred to a new Eppendorf tube and centrifuged again at 20000 rcf for 20 min at 4°C. Finally, after discarding the pellet, the supernatant was used directly in the immunoprecipitation with the Nbea antibody.

Preparation of brain subcellular fractions

Hippocampi were homogenized in ice-cold homogenization buffer (0.32 M sucrose, 5 mM HEPES, pH 7.4) containing protease inhibitor (Roche Applied Science). Cell debris and nuclei were removed by 1.000 g centrifugation for 10 min. The supernatant was either spun at 12.000 g for 20 min resulting in supernatant and pellet P2 (crude membrane; synaptosomes, mitochondria and myelin) or at 100.000 g for 2 hours to obtain a fraction enriched in crude membranes with microsomes (pellet2+microsomes).

The P2 pellet was fractionated by centrifugation (100.000 g, 2 hrs) in a sucrose step gradient to purify synaptosome, an organelle that contains both pre- and postsynaptic compartments. Synaptosome was lysed in hypotonic solution to release the cytoplasmic proteins and organelles such as mitochondria and small synaptic vesicle, and the resulting synaptic membrane was recovered by centrifugation using the sucrose gradient as stated above.

Further synaptic membranes were mixed with an equal volume of 2% Triton X-100, 5 mM HEPES, pH 7.4 containing protease inhibitor and stirred for 30 min over ice. Obtained lipid rafts and PSD were recovered on a sucrose gradient consisting of 1 M, 1.5 M and 2 M sucrose. Lipid rafts were collected at the top of 1 M sucrose and PSD at interphase 1.5 M and 2 M.

Sample preparation for Mass Spectrometry

The ID PAGE LC-MS/MS analysis of protein was carried out as described previously [43]. In short, the immuno-isolated samples were resuspended in reducing SDS loading buffer and heated for 5 min 98 °C before being separated on a 8% SDS polyacrylamide gel. After coomassie staining the sample lane was cut into five slices, each was subjected individually to trypsin digestion. The slices were cut into small pieces and washed for 20 min at room temperature sequentially with 600 µl 25 mM NH_4_HCO_3_/50 % acetonitrile, 100 % acetonitrile and 25 mM NH_4_HCO_3_, the whole cycle was repeated three times; except the last one, which was stopped at the washing step with 100 % acetonitrile. The gel pieces were dried in a Speedvac for 30 min and afterwards rehydrated with trypsin solution. About 200 µl of extra 25 mM NH_4_HCO_3_ was added to each slice, and then incubated at 37 °C over night. The supernatant containing tryptic peptides were transferred to a new tube. Peptide mixtures were dried in a Speedvac and subsequently stored at -20°C until mass spectrometric analysis.

LC-MS/MS analysis

The peptides were redissolved in 15 µL 0.1% acetic acid, and injected into the loop of a Eksogent nano LC-ultra 1D plus HPLC system equipped with a C18 column (200-mm homemade Alltima C18 analytical column, 100 μm ID 3 μm particle size). Peptides were separated using a linear gradient of 5 % solvent A (0.1 % acetic acid, 5 % acetonitrile) and 45 % solvent B (0.1 % acetic acid, 80 % acetonitrile) in 50 min. The LC system was directly coupled in-line with a LTQ-Orbitrap Velos instrument (Thermo Fisher Scientific).

The LTQ-Orbitrap was set to data dependent mode to switch automatically between MS and MS/MS. MS spectra range from 330 till 2000 m/z can be acquired in the Orbitrap at a FWHM resolution of 30,000 after accumulation to 500,000 in the linear ion trap with one microscan. The three most abundant precursor ions were selected for fragmentation by CID, with an isolation width of 2 DA. CID was performed in the linear ion trap after accumulation to 50,000 with 1 microscan.

**Primers used to create Nbea truncations and point mutations**

In order to create the GFP-Duf-PH-BEACH (encompassing AA1956-2553 of Nbea), the following primers were used: RZ550 5’AAACTCGAGGAAGGAAGGTTGTTGTGCCATG C3’ and RZ111r 5’TTTGGATCCTCACCTAGGCGGATGTGGCTCAATGAGC3’. For the GFP-PH-BEACH-WD40 (AA 2140-2925) RZ562 5’AAACTCGAGGGCCCTGTGGTTCTCA GCACC3’ and RZ549r 5’TTTGAATTCTCAATCTATATTAAAAGCTACAATGCTGCC3’ were used. For the GFP-PH-BEACH (AA 2140-2553) RZ90 5’AAAGAATTCAGGGCCCT GTGGTTCTCAGCACC3’ and RZ87r 5’CCTAGGCGGATGTGGCTCAATGAGC3’ were used. For the GFP-PH (AA 2140-2247) RZ90 and RZ88r 5’AACCCGAGGCAAGCTGTA GAC3’ were used. For GFP-BEACH (AA2215-2553) the primers RZ91 5’AAAGAATTCA CACTCAGCGCTGGCAAAGAAGGG3’ and RZ87r were used.

The point mutations were created using the following primers:

E2090K 5’GCACTGCTCAAATCTGCAGTAAAGTATGGCACTGAAGAAGATGTG3’ and E2090Kr 5’CACATCTTCTTCAGTGCCATACTTTACTGCAGATTTGAGCAGTGC3’, E2218R 5’CTTCTGCAGAACACTGCGCTGAGAGTGTTCATGGCGAACCGAACC3’ and E2218Rr 5’GGTTCGGTTCGCCATGAACACTCTCAGCGCAGTGTTCTGCAGAAG3’, N2302A 5’GCAGGTCGGACGTATGCTGATCTGAACCAGTATCCTGTG3’ and N2302Ar 5’CACAGGATACTGGTTCAGATCAGCATACGTCCGACCTGC3’, V2773I 5’GTCCTCACAGGCCATGACCATGAAATTGTCTGTGTCTCCGTC3’ and V2773Ir 5’GACGGAGACACAGACAATTTCATGGTCATGGCCTGTGAGGAC3’, V2346Q 5’GCTTTGAACCCGAAGAGAGCACAGTTTTACGCAGAGCGCTATG3’ and V2346Qr 5’CATAGCGCTCTGCGTAAAACTGTGCTCTCTTCGGGTTCAAAGC3’, E2447R 5’GTACCATCTTGGAGTGAGGAGGGACGAAGTGGTGGTTAATG3’ and E2447Rr 5’CATTAACCACCACTTCGTCCCTCCTCACTCCAAGATGGTAC3’, P2499S 5’GGCTACAAACAGCGAGGGTCAGAGGCAGTCCGTGCTCTC3’ and P2499Sr 5’GAGAGCACGGACTGCCTCTGACCCTCGCTGTTTGTAGCC3’.

**Calcium phosphate transfection of HEK293T cells**

For one 10 cm dish of HEK293T cells (80% confluency) a total of 10 µg of DNA (5µg of each construct) was mixed with 400 µl of 2x Hepes-buffered saline (HBS; containing: 140 mM NaCl, 1.5 mM Na2HPO4.2H2O, 50 mM HEPES adjusted to pH 7.1 with NaOH). Under constant vortexing, 400 µl of 2M CaCl_2_ solution was added dropwise and let to precipitate for 30 min. at RT. Afterwards, this mixture was pipetted in a dropwise-manner onto the plated cells, which were subsequently placed back into the incubator at 37°C for 17 hrs. Next, the medium was replaced with warm fresh culture medium and incubation was resumed for another 24 hrs prior to assaying.

**Confocal imaging and quantification of transfected HEK293T cells**

For confocal imaging cells were transfected as described above and subsequently fixed in 3.7% formaldehyde (Electron Microscopy Sciences) in Dulbecco’s phosphate buffered saline (D-PBS; Gibco) for 20 min. Afterwards cells were washed with D-PBS, mounted on microscopic slides with ProLong®Gold (Invitrogen) and imaged with a Zeiss 510 Meta Confocal microscope. To determine the fraction of cells exhibiting a compartmentalized expression pattern, images of 5 different fields of view were analyzed. In every field of view first all the cells expressing the corresponding construct (regardless of the pattern of expression) were counted and then the cells showing the compartmentalized pattern were counted and the fraction of these cells in regard to the total number of expressing cells was calculated. The fractions in the bar graph in Figure S5B represent the average fractions from the 5 different fields of view.
